# Proteomics-based approach reveals the involvement of spliceosomal components SF3B and SerpinB9 in dermatofibrosarcoma protuberans

**DOI:** 10.1186/s13023-026-04458-4

**Published:** 2026-06-26

**Authors:** Yang Xie, Haifeng Li, Tian Liu, Lingshuo Wang, Mei Yang, Miaojian Wan, Jingang Huang, Yafeng Zhu

**Affiliations:** 1 Department of Dermatology, The Third Affiliated Hospital of Sun Yat-Sen University, Guangzhou, China; 2 Department of Dermatology, The First People’s Hospital of Kashgar Region (Sun Yat-Sen University Affiliated Kashgar Hospital), Kashgar Prefecture, China; 3 Department of Pathology, The Third Affiliated Hospital of Sun Yat-Sen University, Guangzhou, China; 4 State Key Laboratory of Oncology in South China, Sun Yat-Sen University Cancer Center, Guangzhou, China; 5 Guangdong Provincial Key Laboratory of Malignant Tumor Epigenetics and Gene Regulation, Guangdong-Hong Kong Joint Laboratory for RNA Medicine, Medical Research Center, Sun Yat-Sen Memorial Hospital, Sun Yat-Sen University, Guangzhou, China

**Keywords:** Dermatofibrosarcoma protuberans, Proteomics, Splicing, SF3B, SerpinB9

## Abstract

**Background:**

Dermatofibrosarcoma protuberans (DFSP) is a rare cutaneous soft tissue sarcoma which is prone to high recurrence rate, and the fibrosarcomatous transformation of DFSP (FS-DFSP) is associated with a poorer prognosis and significant metastatic potential. Although the translocation of regions of chromosomes 17 and 22 that results in COL1A1-PDGFB fusion is generally acknowledged as a key genetic feature of DFSP, there is no established salvage treatment following resistance to imatinib, which targets the PDGFB pathway. In this study, we performed quantitative proteomics analyses of DFSP tumor tissues and paired adjacent normal skin, to uncover novel molecular mechanisms and potential therapeutic targets. Findings were validated using immunohistochemistry, Western Blot, Real-Time Quantitative PCR and cell viability assay.

**Results:**

Pathway enrichment analysis including KEGG and GSEA revealed that spliceosome pathways and ECM-receptor interaction were associated with DFSP pathogenesis. The spliceosomal components SF3B, and SerpinB9, which is the endogenous inhibitor of granzyme B, were validated to be upregulated in tumor tissues. SerpinB9 was significantly overexpressed in the FS-DFSP subtype compared with the classic-type DFSP. Treatment with the SF3B1 inhibitor, Pladienolide-B, significantly reduced tumor cell viability. siRNA-mediated knockdown of SF3B1 and SF3B2, as well as Pladienolide-B treatment, inhibited *SERPINB9* expression without affecting pre-*SERPINB9* mRNA levels, suggesting splicing-dependent regulation. Additionally, quantitation of the tumor microenvironment cells using the MCP-counter algorithm indicated NK cells activation. The CD56 (NK cell surface marker), biomarkers of tumor-associated macrophage (TAM) such as CD14 and CD163, and transforming growth factor-β induced (TGFBI) which is associated with TAM infiltration stained positive in DFSP tumors. *SERPINB9* mRNA expression showed a positive correlation with both NK cell abundance and FCGR3A (NK cell marker) mRNA level.

**Conclusions:**

SerpinB9 overexpression is associated with fibrosarcomatous features in DFSP and may represent a candidate prognosis biomarker. The SF3B/SERPINB9 axis is a potential therapeutic vulnerability, particularly in FS-DFSP. NK cell- and macrophage-related signatures were evident in DFSP tumor microenvironment, warranting further functional studies.

**Supplementary information:**

The online version contains supplementary material available at 10.1186/s13023-026-04458-4.

## Background

Dermatofibrosarcoma protuberans (DFSP) is a relatively rare sarcoma, constituting approximately 1.1% of soft-tissue tumors, with an annual incidence rate of around 4.2 cases per million [1, 2]. DFSP is a locally aggressive and invasive neoplasm with a high recurrence rate due to its infiltrative behavior. The median age of diagnosis typically falls between 20 and 59 years old, although congenital cases and pediatric patients are not uncommon [3]. Skin injury at the lesion site is one of the potential risks of DFSP. In addition, DFSP may exhibit accelerated growth during pregnancy [4, 5].

Histopathologically, DFSP is characterized by uniform spindle cell fascicles growing in a storiform pattern with multiple variants, exhibiting strong and diffuse CD34 immunoreactivity. While most of the DFSP tumors are low-grade lesions, sarcomatous transformation from the classic type occurs in 10–20% of cases. The fibrosarcomatous DFSP (FS-DFSP) subtype is the most aggressive and considered to have higher rates of local recurrence and metastasis [1]. It is noteworthy that the risk of transformation increases with the recurrence times.

While chromosome t(17;22) translocations leading to COL1A1-PDGFβ fusion represent a known genetic driver which occurs in more than 90% of cases, diverse alternative fusions (e.g., t(2;17), t(9;22), t(5;8)) highlight molecular heterogeneity [6]. The t(17;22) leads to the replacement of PDGFβ exon1 by COL1A1 elements forming a fusion oncogene COL1A1-PDGFβ. The tyrosine kinase inhibitor imatinib mesylate, which targets the PDGFβ pathway, is the only drug recommended for the unresectable or metastatic DFSP [1, 7]. It has been reported that patients undergoing post-imatinib resection subsequently relapse, suggesting that metastatic disease cannot be eradicated by imatinib strategies. However, no effective salvage therapies exist after imatinib failure or resistance, underscoring a critical gap in DFSP management.

The etiology of DFSP progression remains poorly understood, necessitating comprehensive proteomic analyses to uncover novel molecular mechanisms and therapeutic targets. Here, we performed quantitative proteomic profiling of tumor tissues versus paired adjacent non-tumor (ANT) skin samples from DFSP patients to discover potential novel therapeutic targets.

## Methods

### Clinical samples collection

Patients of DFSP were treated with Mohs micrographic surgery in the department of dermatology, the Third Affiliated Hospital of Sun Yat-sen University, during November, 2019 to October, 2025. All patients were adults and diagnosed as DFSP by one general pathologist and one dermatopathologist. As Mohs micrographic surgery enables margin assessment and achieves complete R0 resection, the peritumoral tissues collected in this study were considered histologically normal non-tumor tissues. Corresponding histopathological data from formalin-fixed paraffin-embedded (FFPE) sections were available and matched for all samples.

Five cases (patients#1-#5) of the tumor tissues and ANT skin tissues were used for proteomic analysis, immunohistochemical (IHC) stain, and Real-Time Quantitative PCR (RT-qPCR). To minimize the error, the five cases of classic type DFSP were male who had a single tumor on the back. In order to validate the results of proteomic analysis, tumor tissues or primary cells of additional 28 DFSP cases were collected for IHC, Western Blot and RT-qPCR. The demographic and clinicopathological features of the DFSP patients from whom tissue samples/primary cells were obtained and the experimental procedures performed on these materials are listed in Additional file 1.

### Proteomics sample preparation

To minimize protein degradation, all tumoral and peritumoral skin specimens obtained from the five patients were frozen in the liquid nitrogen 30 minutes after surgery. For the peritumoral skin tissues, visible subcutaneous fat was trimmed away. Tissue samples were washed three times with phosphate-buffered saline (PBS) buffer. Samples were minced and lysed in a buffer containing 8 M urea, 50 mM Tris-Cl, and 1 X protease inhibitor, then sonicated for 2 minutes (3 s on, 2 s off, 40% amplitude). The lysates were centrifuged at 20,000 × g for 15 minutes, and supernatants were collected. The protein concentration was determined by BCA protein quantitative kit. An aliquot of 50 µg protein solution 50 (adjusted to 1 µg/µL) was transferred to a fresh tube. DTT was added to a final concentration of 10 mM and incubated at 56 °C for 1 hour. After cooling to the room temperature, IAA was added to a final concentration of 55 mM and incubated in the dark at room temperature. The solution was then diluted with 50 mM ammonium bicarbonate (ABC, pH 8.0) until urea concentration was below 1 M. Trypsin (1 µg) was added, and the digestion was incubated at 37 °C for 12 hours. On the following day, the equal amount of trypsin (1 µg) was added, and incubation was continued for another 4 hours. The enzymatic digestion was terminated by adding 20% trifluoroacetic acid (TFA) to a final concentration of 1% TFA. The tube was centrifuged at 14,000 g for 15 minutes at room temperature, and the liquid in the collection tube was transferred to a new centrifugal tube for vacuum drying. Finally, the samples were desalted using C18 desalting column.

### Mass spectrometry analysis

The chromatographic mobile phases consisted of 0.1% formic acid (FA) in water (buffer A) and 80% acetonitrile (ACN) with 0.1% FA (buffer B). The lyophilized peptide was fully dissolved in 0.1% FA and centrifuged at 17,000 g for 15 minutes. The supernatant was transferred to the sample vial and placed in the automatic sampling device. Easy nano 1200 liquid chromatography (Thermo, USA) was performed at a flow rate of 3 μL/min from the automatic injector into the C18 analytical column (internal diameter 150 μm, 30 cm) for elution. Liquid chromatography was performed at a flow rate of 600 nL/min under the condition that liquid B rose linearly from 7% to 12% between 0 and 8 minutes, 12% to 30% between 8 and 90 minutes and 30% to 45% till 110 min. Thermo Scientific Orbitrap Exploris 480 mass spectrometer and Nanospray Flex ion source were used to set the ion spray voltage at 2.2 kV and the ion transport tube temperature at 320 °C. A FAIMS Pro interface was operated at two compensation voltages (−45 V and −65 V), with each cycle time set to 1 second. For MS1, the scanning range was 350–1500 m/z, the resolution was 60 K, the AGC target was 3e6, Maximum injection time (IT) was 50 ms. For MS2, the resolution was 15 K, Isolation window 1.6Th, AGC target 7.5e4, maximum IT 22 ms, MS2 Activation HCD (collision) energy was set to 30. The MS raw data was analyzed by Proteome Discoverer Software (V2.4) and the built-in Sequest HT for analysis. Specific Settings: Fixed modification = Carbamidomethylation (C); Variable modifications: Acetyl (Protein N-term) and Oxidation (M); The false-positive rates (FDR) of peptide and protein were set at 1%. All other parameters were default. The MS1 precursor mass error tolerance was set to 10 ppm, and the mass tolerance of MS2 fragment ion was set to 0.05 Da. The protein database downloaded from Uniprot was used for database searching [8].

### Gene-disease association network and pathway enrichment Analysis

Bioconductor package *ClusterProfiler* was used for gene-disease association and KEGG pathway enrichment analysis [9]. Adjusted *p*-value < 0.05 was statistically significant, and the analytical results were visualized. GSEA Pre-ranked analysis was performed on genes ranked by log2FC against MSigDB KEGG and Reactome, with significance set at FDR q < 0.25. Enrichment plots were generated for visualization.

### IHC stain

The expression of Tenascin C (TNC), fibroblast activation protein-α (FAP), CD14, CD163, and CD56 was evaluated by immunohistochemical (IHC) detection using corresponding primary antibodies. Specifically, rat monoclonal antibodies against TNC (Abcam, #ab108930, Cambridge, UK), FAP (Abcam, #ab207178), CD14 (Abcam, #ab133335), and CD163 (Abcam, #ab182422) were used, while a mouse monoclonal antibody against CD56 (clone CD564, Quanhui Commercial Co., LTD., Guangzhou, China) was employed. Antibodies against transforming growth factor-β induced (TGFBI) and serine protease inhibitor B9 (SerpinB9) were obtained from Zhengneng Biological Technology (#R380652, Chengdu, China) and Proteintech Group (#67950–1-Ig, Chicago, USA), respectively. SerpinB9 staining intensity was quantified using the IHC Toolbox plugin of ImageJ software. For each tumor section, at least five fields of view (magnification, ×200) were counted, and the average staining intensity was calculated for subsequent analyses.

### Protein extraction from human tissues

Total protein was extracted from human tissues using RIPA Lysis Buffer (Strong, CW2333s, CWBIO). Briefly, 100 mg of each tissue sample was placed into a 2 ml pre-cooled EP tube with 300 µl RIPA lysis buffer (CW2333s, CWBIO) supplemented with 1 mmol/L Protease inhibitor Cocktail (CW2200s, CWBIO) and 1 mmol/L Phosphatase inhibitor Cocktail (CW2383s, CWBIO) along with 2 grinding beads (G0103-200 G, Servicebio). Then, the tubes were put into the pre-cooled freeze grinder (JXFSTPRP-CL) (−10 °C) and homogenization was performed with the following program: 70 HZ, 60 s of grinding followed by 30 s of rest, for 6 cycles (normal control samples) or 3 cycles (tumor samples). After homogenization, the samples were lysed on ice for 30 minutes and centrifuged at 12,000 rpm for 30 minutes with the grinding beads removed prior to centrifugation. The supernatant was transferred to a fresh microcentrifuge tube and subjected to a second centrifugation at 12,000 rpm for 10 min. The final supernatant was collected and stored at − 80 °C until further use.

### Western-Blot Analysis

Protein concentration was quantified by the BCA method according to the manufacture’s protocol (CW0014s, CWBIO). Equal amounts of protein (40 µg per lane) were loaded onto 10% SDS-PAGE gels and transferred onto PVDF membrane. Then, membranes were blocked with 5% non-fat milk (Blotting Grade, P0216) for 1 h, incubated with indicated primary antibodies overnight at 4 °C, washed with PBST for 3 times with 10 minutes each, and incubated with HRP-conjugated secondary antibodies for 1 h at room temperature. Finally, chemiluminescence signals were detected using a SmartChemi 910 Plus imaging system (Sagecreation) with an enhanced chemiluminescence reagent (FDbio-Pico ECL Kit, FD8000). Primary antibodies including SF3B1 (#ab172634, Abcam), SF3B2 (#10919–1-AP, Proteintech) and GAPDH (#RM2002, Ray Antibody), as well as secondary antibodies including goat anti-mouse IgG HRP (#HS201-01, TransGen Biotech) and mouse anti-rabbit IgG HRP (#sc-2357, Santa Cruz) were used in this experiment. The dilution ratios were 1:1000 for SF3B1 and SF3B2 primary antibody, 1:5000 for GAPDH primary antibody, and 1:5000 for all secondary antibodies.

### Primary cell culture

Primary DFSP cells were prepared from tumor tissues obtained during surgery. Human dermal fibroblasts (HDF) were obtained from the pediatric foreskin tissue following circumcision. Tissues were aseptically isolated and minced in ice-cold HBSS supplemented with 1% antimycin. Then the tissues were cut into small pieces and enzymatically dispersed by treatment with collagenase D (Roche) at 37 °C overnight. The suspension was washed with DMEM twice and placed on a 100-μm nylon cell strainer. Cells were passed through the strainer and were cultured in DMEM supplemented with 10% fetal bovine serum (FBS; ExCell Bio), 100 U penicillin G, and 100 μg/mL streptomycin (Gibco). In addition, 4 μM of ROCK inhibitor Y-27632 (MCE) was added in the culture medium to maintain tumor stemness. Cells were seeded in a T25 culture dish and maintained at 37 °C in a humidified atmosphere of 5% CO_2_ incubator.

### Pladienolide B treatment

Pladienolide B (PLB) (#GC18780, GLPBIO Technology Inc, Montclair, USA) was dissolved in DMSO at 10 mM and stored at −20 °C. For use, the inhibitor was diluted in culture medium to final working concentrations. For cell viability assays, DFSP primary cells were treated with PLB at concentrations ranging from 0 to 100 nM for 72 hours. Cell viability was then assessed using the CCK-8 assay. For further qPCR experiments, DFSP primary cells were plated in 6-well plates, and drugs were added when cells reached ~80% confluence. After incubating for 72 h, cells were lysed and subjected to qPCR analysis.

### RNA extraction

For RNA extracted from formalin-fixed paraffin-embedded (FFPE) tissue blocks, a commercial nucleic acid extraction kit (Amoy Diagnostics, Xiamen, China) was used. For RNA extraction of primary cells, cells were rinsed with cold PBS twice, then total RNA was extracted using RNA Easy Fast Tissue/Cell Kit (TIANGEN, DP451), according to the manufacturer’s protocol.

### RT-qPCR

First-strand complementary DNA (cDNA) was generated by PrimeScript™ RT Master Mix (Takara, #RR036A) using 500 ng of RNA, following the manufacturer’s protocol. cDNAs were stored at − 20 °C for further usage. Gene expression was determined by RT-qPCR with TB Green® Premix Ex Taq™ II (Tli RNaseH Plus) (Takara, #RR820A) using LightCycler® 480 II Real Time PCR System (Roche, 96-well). The 2^−∆∆CT^ method was applied to determine the relative gene expression.

### siRNA assay

To achieve targeted knockdown of SF3B1 and SF3B2, small interfering RNAs (siRNAs) specific to each gene were employed. The siRNA sequences for SF3B1 (sense: 5′-GGUCUGGCUACUAUGAUCUTT-3′; antisense: 5′-AGAUCAUAGUAGCCAGACCTT-3′) and SF3B2 (sense: 5′-GGAACCAUCUGAUGAAGAATT-3′; antisense: 5′-UUCUUCAUCAGAUGGUUCCTT-3′) were obtained from GenePharma (Shanghai, China). A negative control siRNA (NC-siRNA) was used as the control. For transient transfection, cells were seeded in 6-well plates and cultured until reaching 60–80% confluence. Transfection was then performed using the siRNA mate plus transfection reagent (GenePharma) with siRNAs at a final concentration of 75 nM, following the manufacturer’s protocol. At 48 hours post-transfection, cells were harvested for subsequent mRNA and protein analyses.

### Statistical analysis

All statistical tests were performed in R4.0.2 software. The differential protein expression analysis of DFSP proteomics data was performed by DEqMS [10]. The tests performed included paired t-test and ANOVA. The *p* value < 0.05 was considered to be statistically significant.

## Results

### Proteomic analysis revealed a distinct protein profiling between DFSP and ANT

**Fig. 1:**
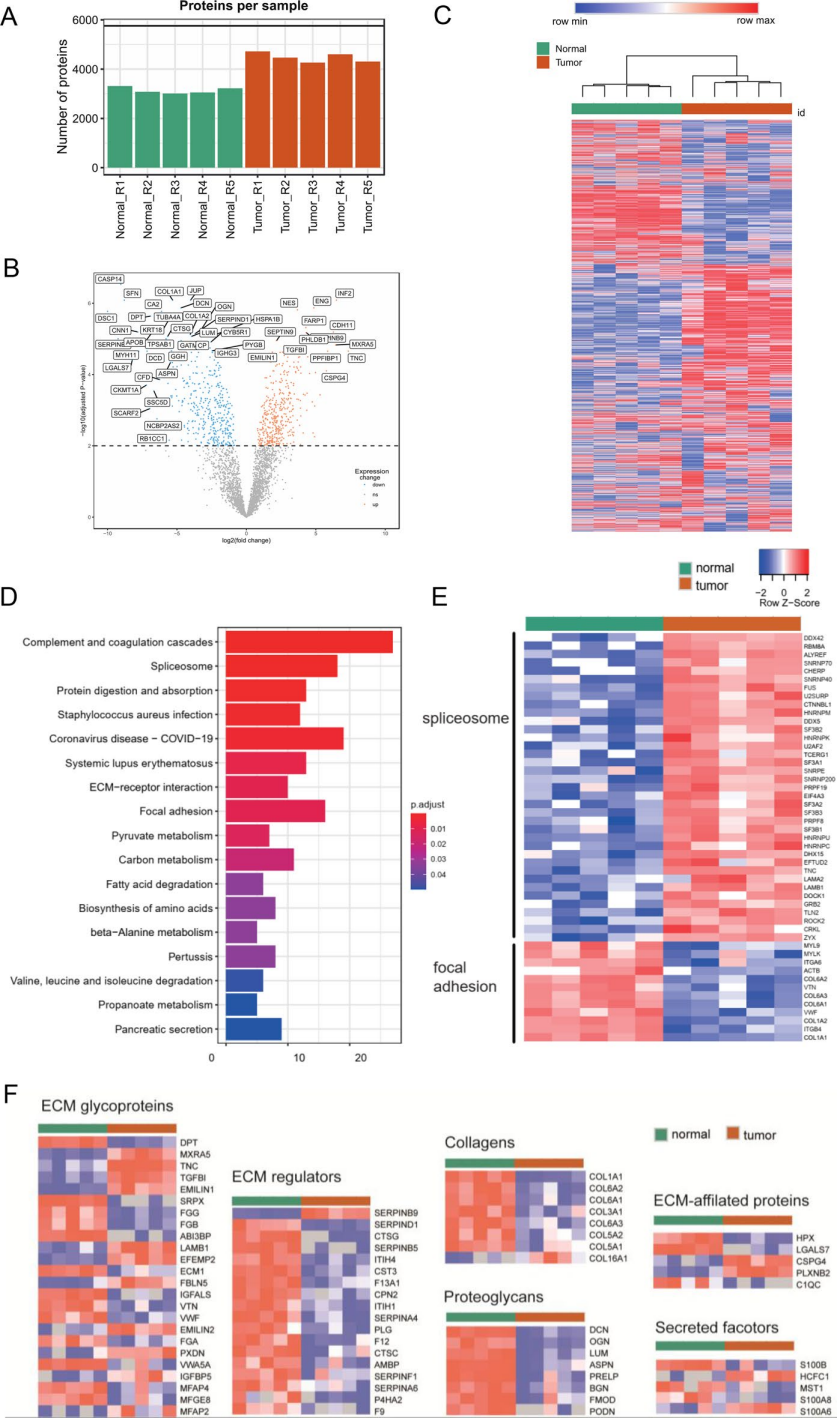
Proteomic analysis for DFSP. (**A**) The bar chart showing numbers of protein identified in each normal (green, *n* = 5) and DFSP tumor (orange, *n* = 5) tissues (patients #1-#5). (**B**) The volcano plot displaying differentially expressed proteins in the DFSP group with adjusted *p* value < 0.01 (based on DEqMS analysis). (**C**) The heatmap plot depicts dysregulated proteins between control group (normal) and DFSP group (tumor). (**D**) KEGG enrichment analysis delineates signaling pathways that are involved in the DFSP. (**E-F**) The heatmap plot depicts dysregulated proteins of enriched pathways shown in panel (**D**). All enrichment and clustering analyses were performed using only the significantly dysregulated proteins, selected with the criteria of |fold Change| >1.5 and FDR < 0.05 (Fig. C-F). Detailed data were provided in the Additional file 2

In total, 5760 proteins were identified from 10 clinical samples (tumor tissues and ANT from patients #1-#5). Figure 1 displays the proteomics profile from the five DFSP patients. On average, 4473 and 3138 proteins are identified from tumor and normal tissues respectively (Fig. 1A). For downstream analysis, we retained 2820 proteins with at least three quantitative values from both tumoral and peritumoral samples.

We applied DEqMS, a proteomics differential analysis tool, and identified 1308 significantly differentially expressed proteins (adjusted *p*-value < 0.05), comprising 589 downregulated and 719 upregulated proteins in tumor samples. (Fig. 1B, Additional file 2). Unsupervised hierarchical clustering analysis demonstrates a clear separation between tumors and ANT (Fig. 1C).

### mRNA splicing and ECM were dysregulated in DFSP

We conducted KEGG enrichment analysis to further characterize the biological pathways of differentially regulated proteins between DFSP tumors and ANT. The results of the KEGG analysis suggests that the differentially expressed proteins (DEPs) are primarily enriched in 17 pathways (Fig. 1D). Among these pathways, the most significant biological processes relevant to the DFSP tumor progression are the spliceosome and the focal adhesions, with genes in spliceosome pathway upregulated and genes in focal adhesions pathway downregulated (Fig. 1E).

We also analyzed the extracellular matrix (ECM)-receptor interaction which was closely related to the focal adhesions. Based on Matrisome DB (http://www.pepchem.org/matrisomedb), we generated expression heatmaps of genes involved in differentially functional groups (Fig. 1F). Most types of collagen and proteoglycans are clearly downregulated in tumors comparing to those in peritumoral normal tissues. For the non-collagen glycoproteins, TNC, TGFBI, laminin Subunit-β1 and EGF-containing fibulin-like extracellular matrix protein 2 are significantly upregulated in all cases of tumors. The ECM regulator SerpinB9 is markedly upregulated in tumors.

**Fig. 2:**
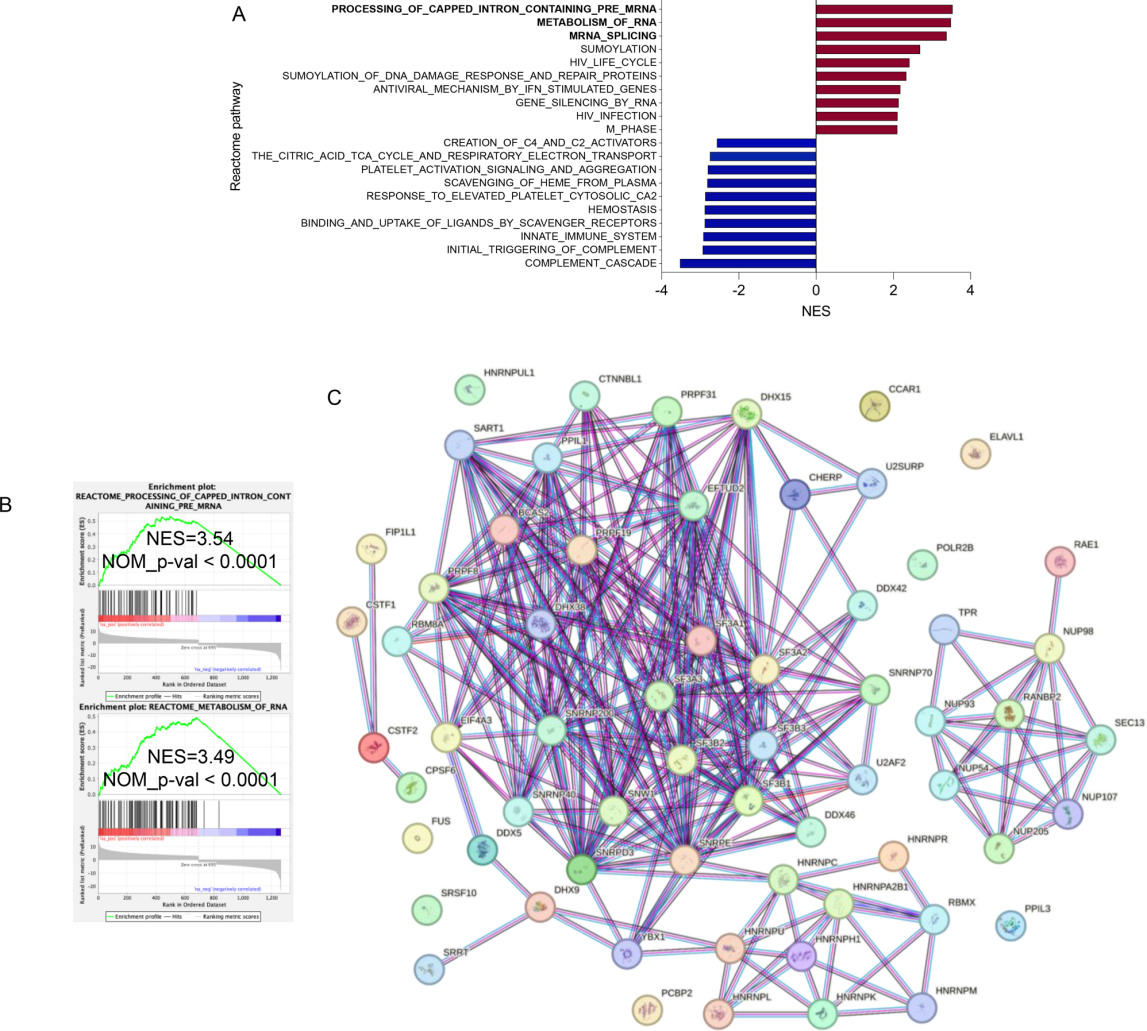
GSEA enrichment analysis and protein-protein interaction (PPI) network. (**A**) GSEA enrichment analysis of DFSP differentially expressed proteins, top 10 positive (red) and top 10 negative (blue) enriched reactome pathways are shown. (**B**) The enrichment plots for mRNA splicing related pathways. (**C**) PPI network of mRNA splicing related pathway

GSEA enrichment analysis of the DEPs was also conducted. The top 10 positive (red) and top 10 negative (blue) enriched Reactome pathways are shown in Fig. 2A. The positively enriched pathways were mainly related to RNA processing and splicing, while the downregulated pathways included citric acid TCA cycle, platelet activation and innate immune response. As shown in Fig. 2B, genes involve in RNA processing are significantly upregulated, while genes in complement cascade pathway are mostly downregulated. The protein-protein interaction (PPI) network of mRNA splicing related pathway are shown in Fig. 2C. In summary, proteomics data revealed that DFSP tumors were characterized by upregulated RNA splicing and disruption of ECM components, e.g. diminished production of collagens and proteoglycans.

**Fig. 3:**
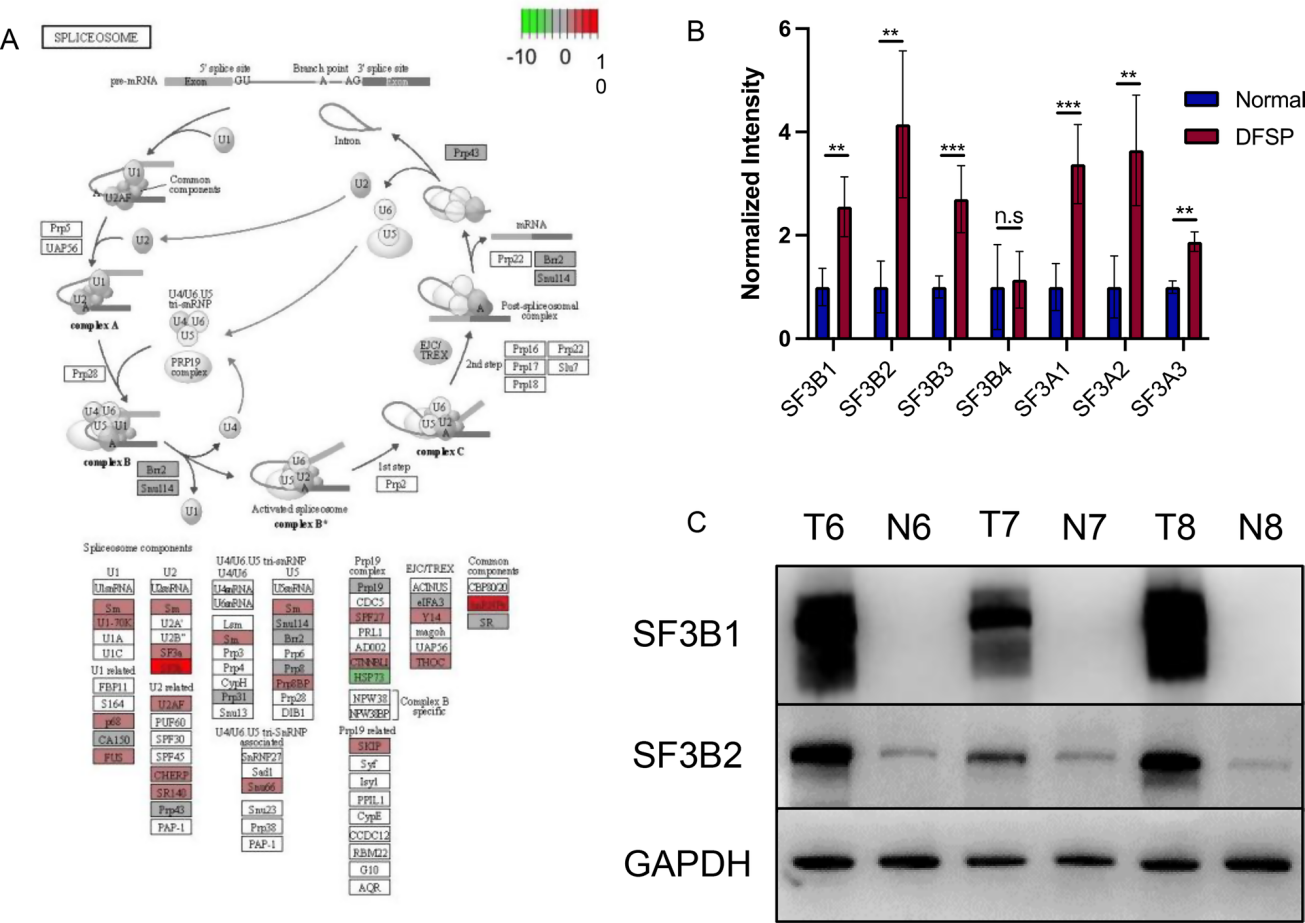
KEGG analysis for spliceosome pathway and and detection of SF3A/B components. (**A**) KEGG analysis of spliceosome pathway. Setting *p* < 0.05 is statistically significant. (**B**) The relative expression of each SF3A/B component involved in mRNA splicing pathways according to KEGG analysis. Data were normalized to average intensity of the normal group and then presented as the mean ± SD. n.S, *p* ≥ 0.05; **, *p* < 0.01; ***, *p* < 0.001. (**C**) Western blot for SF3B1 and SF3B2 between DFSP tumor tissues and adjacent normal tissues from patients #6-#8

To further explore dysregulated genes in the spliceosome pathway, the differentially regulated proteins are visualized in the pathway map (Figs. 3A–3B). Many proteins involved in different spliceosome components were upregulated. In the pre-mRNA splicing process, SF3b protein complex played critical roles in recognition of branch point sequence (BPS) and facilitated spliceosome assembly and activation. To validate upregulation of SF3b complex, western blotting was conducted to examine protein expression of SF3B1 and SF3B2 in tumor tissues from another three DFSP patients #6-#8. Both SF3B1 and SF3B2 expression are higher in the tumor tissues comparing to their ANT (Fig. 3C).

### SerpinB9 was highly expressed in DFSP tumor tissue and positively correlated with tumor subtypes

**Fig. 4:**
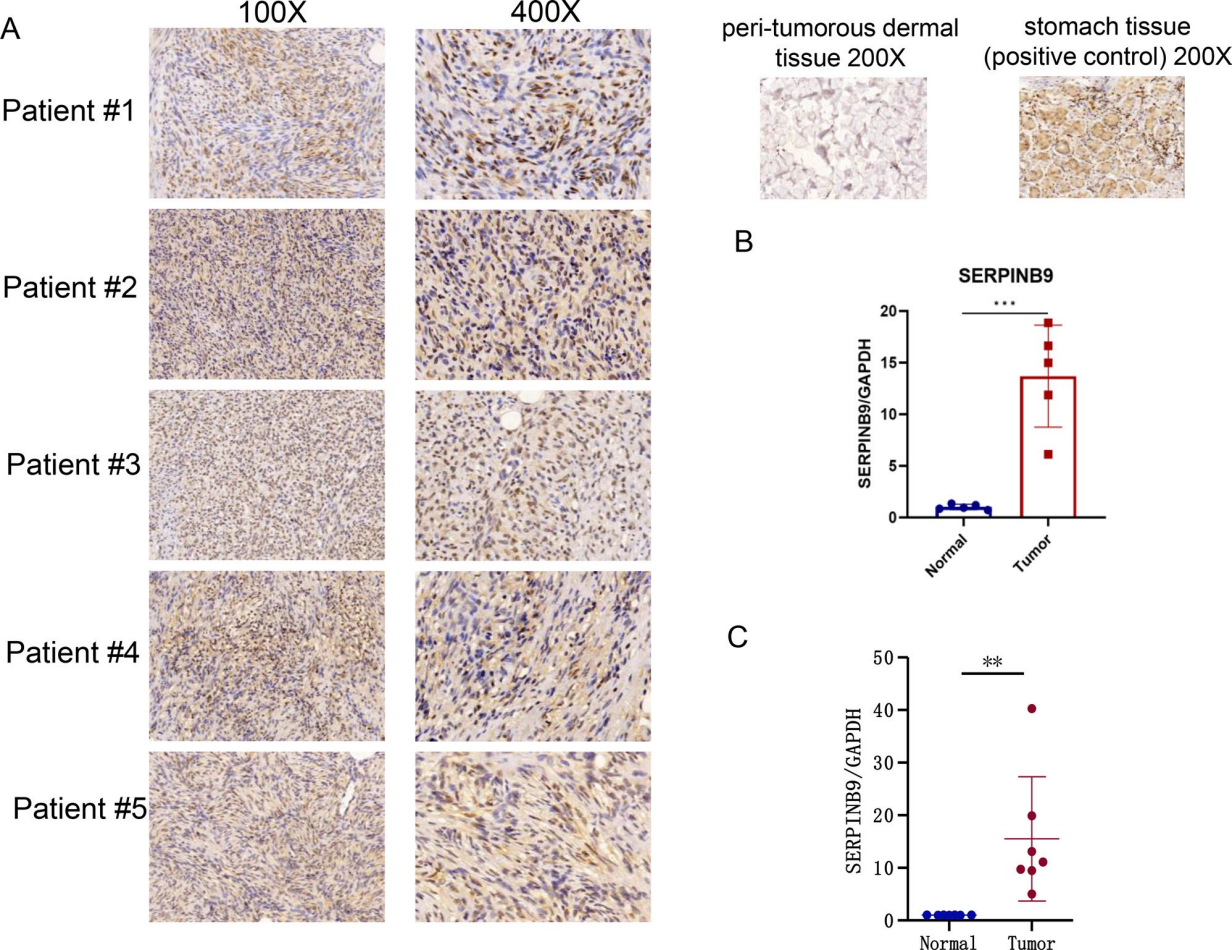
Overexpression of SerpinB9 in DFSP tumor tissues. (**A**) SerpinB9 staining of DFSP tumor tissues from patient #1~#5 and adjacent peri-tumor tissues. SerpinB9 is positive in cytoplasm and nucleus in the DFSP tumor cells and negative in the adjacent peri-tumor tissues by immunohistochemistry. The gastric mucosal tissue is used as positive control. (**B**) RT-qPCR validation for SERPINB9 in DFSP and adjacent peri-tumor tissues from patients #1-#5 (FFPE samples), data represent means ± SD from *n* = 3 replicates. Bars, SD. T-test. ***, *p* < 0.001. (**C**) RT-qPCR validation for SERPINB9 in DFSP and adjacent peri-tumor tissues from patients #6-#12, data represent means ± SD from *n* = 3 replicates. Bars, SD. T-test. **, *p* < 0.01

The upregulated ECM regulator SerpinB9 in DFSP tumor tissues was a physiological inhibitor of granzyme B (GrB). NK cells or cytotoxic T lymphocytes (CTL) usually use the GrB to mediate apoptosis of tumor cells. IHC stain and RT-qPCR for the SerpinB9 were used to validate the proteomic analysis result. Immunohistochemically, the tumor cells from patients #1-#5 are positive for SerpinB9 in the cytoplasm and the nucleus (Fig. 4A). RT-qPCR analysis also shows that *SERPINB9* gene is significantly upregulated in the tumors of patients #1-#5, comparing with their ANT (Fig. 4B). For further validation, we performed RT-qPCR in additional seven cases of classic-type DFSP and obtained consistent results (Fig. 4C, patients #6-#12).

**Fig. 5:**
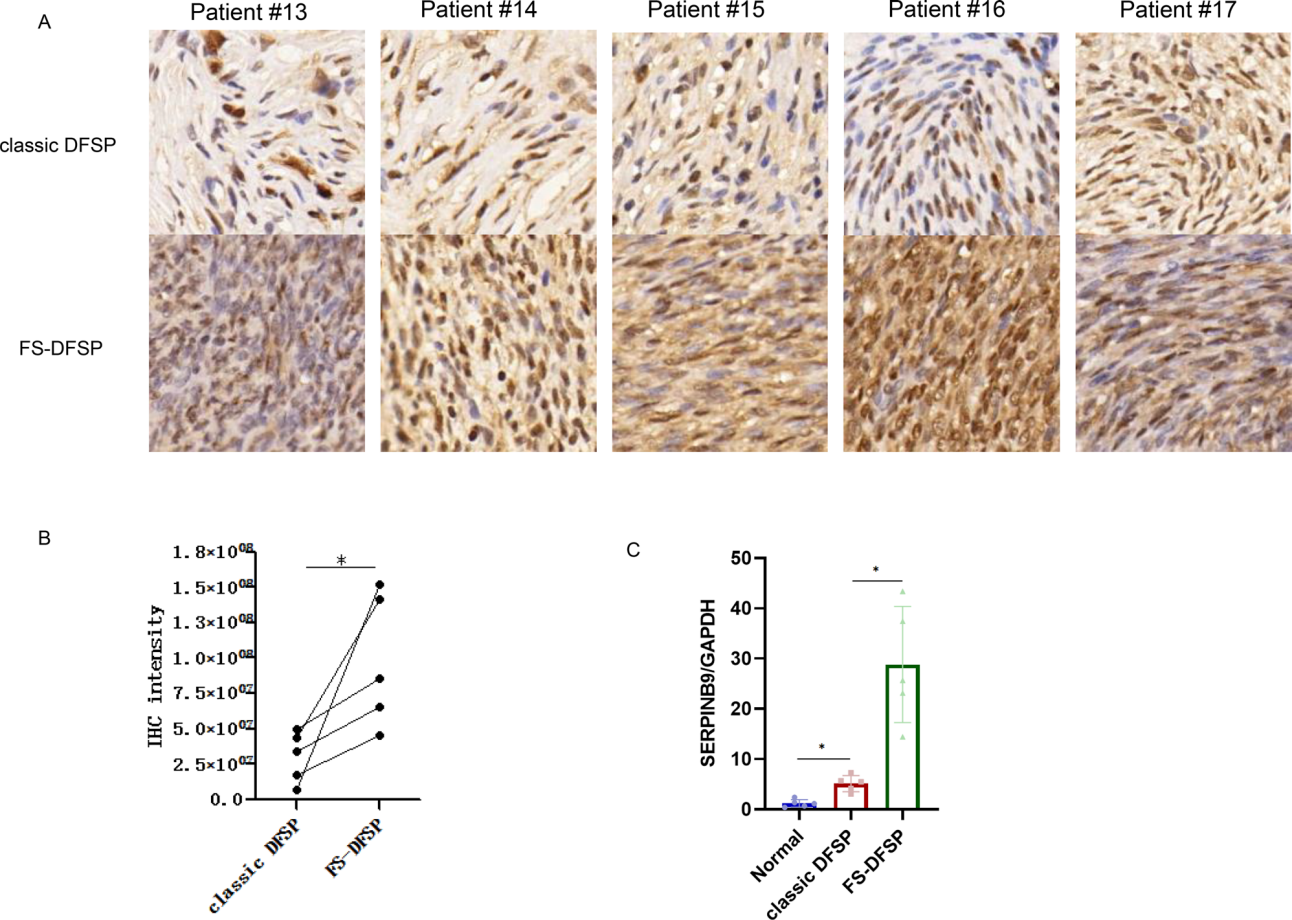
SerpinB9 is significantly overexpressed in FS-DFSP subtype compared with classic DFSP subtype in the same patents. (**A**) The representative pictures of SerpinB9 IHC staining. SerpinB9 staining of classic DFSP and FS-DFSP from patient #13~#17 (400×). (**B**) Statistical analysis of serpinB9 IHC expression intensity analyzed by image J (*, *p* < 0.05). (**C**) RT-qPCR validation for *SERPINB9* in classic DFSP and FS-DFSP, data represent means ± SD from *n* = 3 replicates. Bars, SD. T-test. *, *p* < 0.05. Each paired classic DFSP and FS-DFSP tumor tissues were obtained from the same patient (patient #13-#17, FFPE samples)

To explore the differential expression of SerpinB9 between classic DFSP and FS-DFSP, we quantified SerpinB9 immunohistochemical staining in paired classic and fibrosarcomatous tumor tissues from the same patients (patients #13-#17) with image J software (Fig. 5A and B). Validation is also performed via RT-qPCR (Fig. 5C). Data demonstrated that FS-DFSP exhibited higher expression of SerpinB9 compared with classic DFSP (paired t-test, *p*-value < 0.05).

### Blockade of mRNA splicing inhibited the expression of SerpinB9

**Fig. 6:**
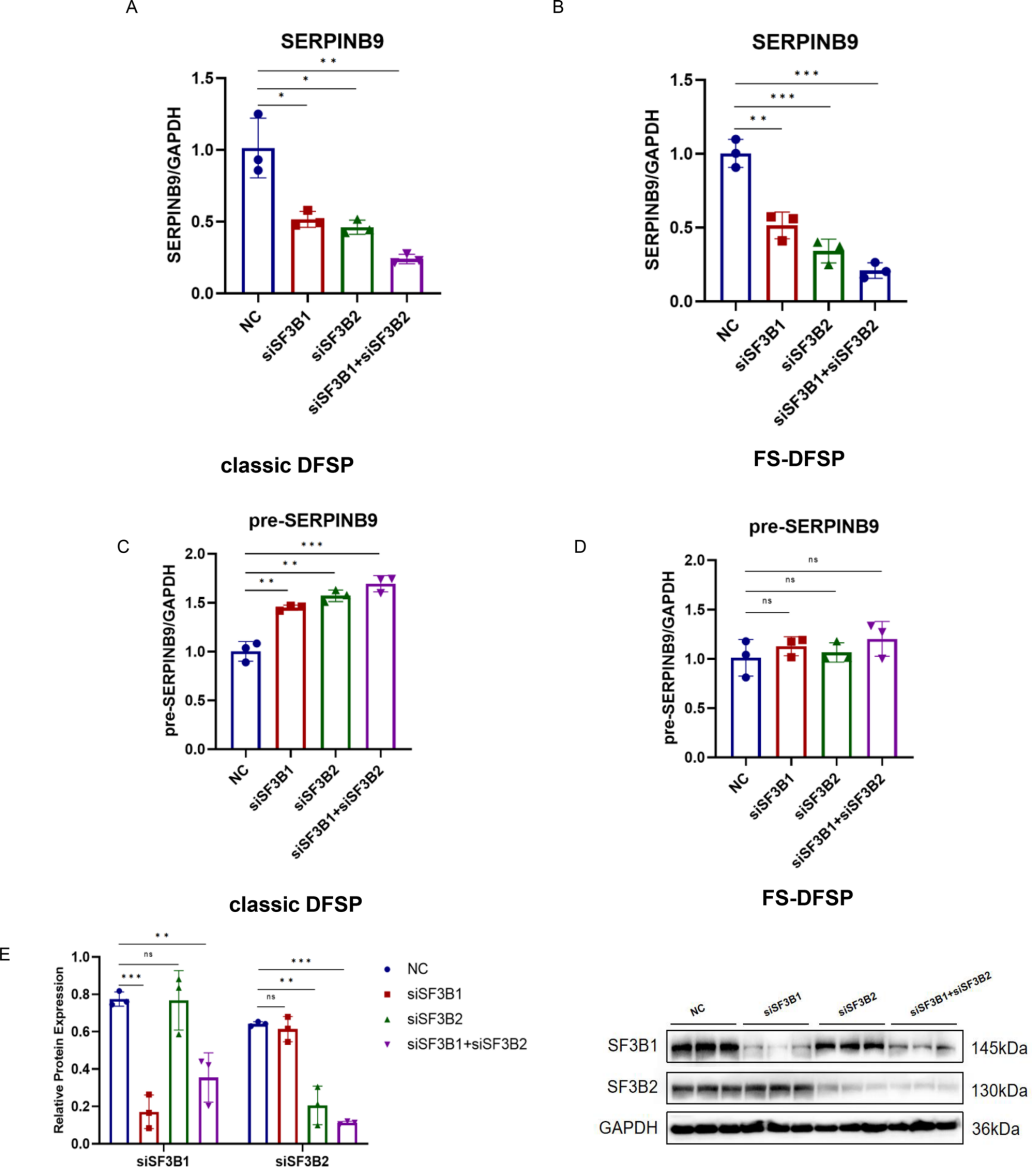
Knockdown of SF3B1/SF3B2 downregulates *SERPINB9* expression but not pre-*SERPINB9* in primary DFSP cells. Cells were transfected with SF3B1 siRNA, SF3B2 siRNA, or negative control siRNA (NC, a non-silencing control). (**A, B**) *SERPINB9* mRNA levels in classic DFSP cells (*n* = 3, patients #18-#20) and FS-DFSP cells (*n* = 3, patients #21-#23). Data represent means ± SD. Bars, SD. ANOVA. *, *p* < 0.05. **, *p* < 0.01. ***, *p* < 0.001. (**C, D**) pre-*SERPINB9* mRNA levels. (**E**) Knockdown efficiency of SF3B1 and SF3B2

**Fig. 7:**
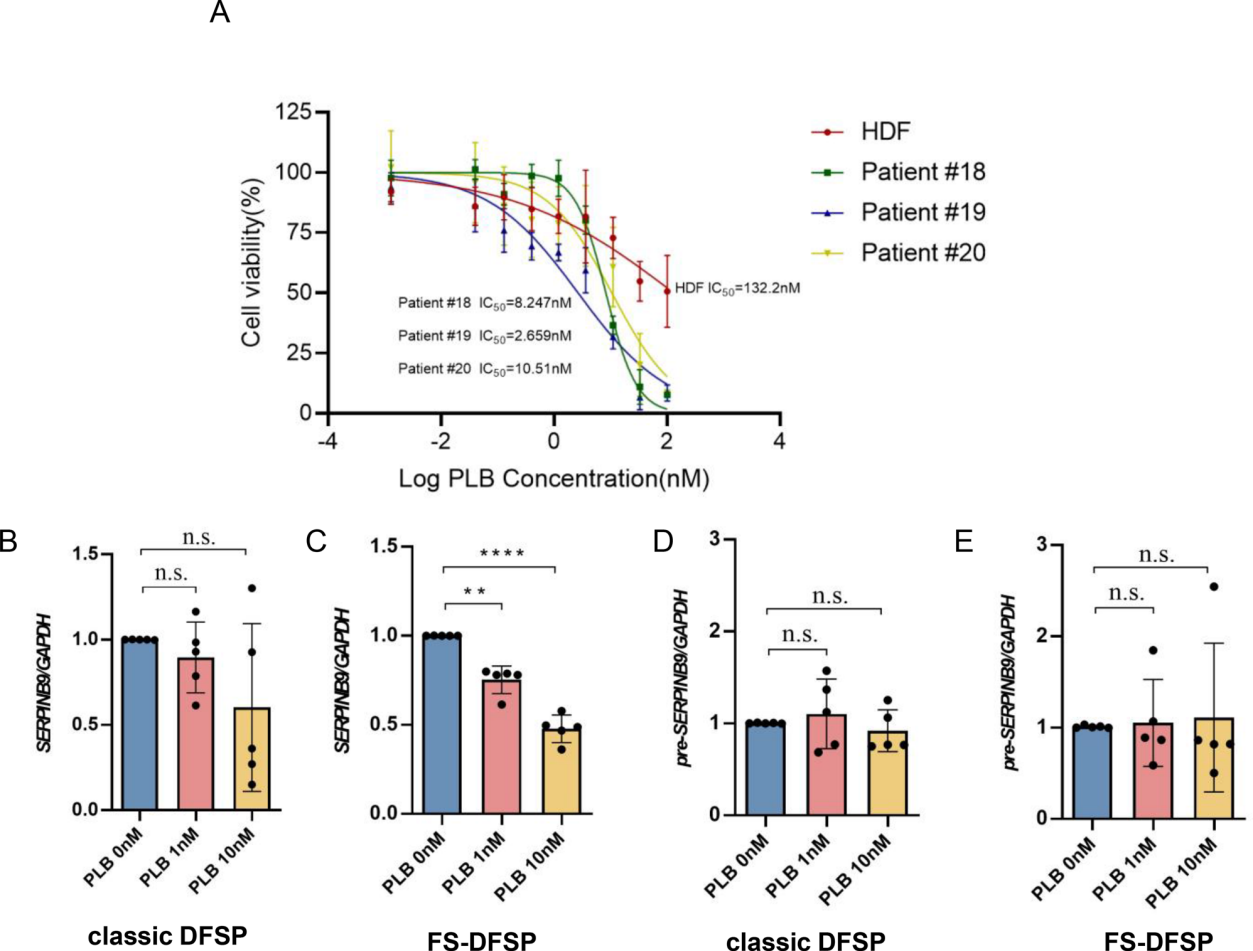
SF3B1 inhibitor PLB reduced cell viability and *SERPINB9* expression but not pre-*SERPINB9* in FS-DFSP tumor cells. (**A**) Following treatment with the SF3B1 inhibitor PLB, the IC50 value of PLB is much lower in DFSP cells than in normal human dermal fibroblasts (HDF) (*n* = 3, patients #18-#20). (**B-E**) RT-qPCR for *SERPINB9* gene and pre-*SERPINB9* in classic DFSP primary cells (*n* = 5, patients #24-#28) and in FS-DFSP primary cells (*n* = 5, patients #29-#33) treated with PLB 1 nM and 10 nM, respectively. Data represent means ± SD. Bars, SD. ANOVA. **, *p* < 0.01. ****, *p* < 0.0001

To investigate whether the expression of SerpinB9 in DFSP is regulated by RNA splicing, we used siRNA-mediated knockdown of SF3B1 and SF3B2, as well as SF3B1 inhibitor PLB to treat the primary cells of DFSP tumors. As shown in Fig. 6, knockdown of SF3B1 and SF3B2 with specific siRNAs significantly downregulates the mRNA expression of *SERPINB9* in primary classic DFSP and FS-DFSP cells (Fig. 6A–6B). In contrast, the expression level of pre-*SERPINB9* is not inhibited upon SF3B1/SF3B2 knockdown (Fig. 6C–D). The knockdown efficiency of SF3B1 and SF3B2 is verified and confirmed (Fig. 6E). Following treatment with the SF3B1 inhibitor PLB, the IC50 value of PLB is much lower in DFSP cells than in normal HDF (Fig. 7A). When treated with PLB, *SERPINB9* expression shows a downward trend in classic DFSP primary cells, but the difference does not reach statistical significance (patients #24-#28, *p*-value > 0.05) (Fig. 7B). On the other hand, *SERPINB9* mRNA level is significantly inhibited in the FS-DFSP primary cells after exposure to PLB (patients #29-#33, *p*-value < 0.01) (Fig. 7C). pre-*SERPINB9* mRNA level is not affected by PLB in either subtype of DFSP primary cells (Fig. 7D–E).

### SerpinB9 expression was positively correlated with NK cells in DFSP

**Fig. 8:**
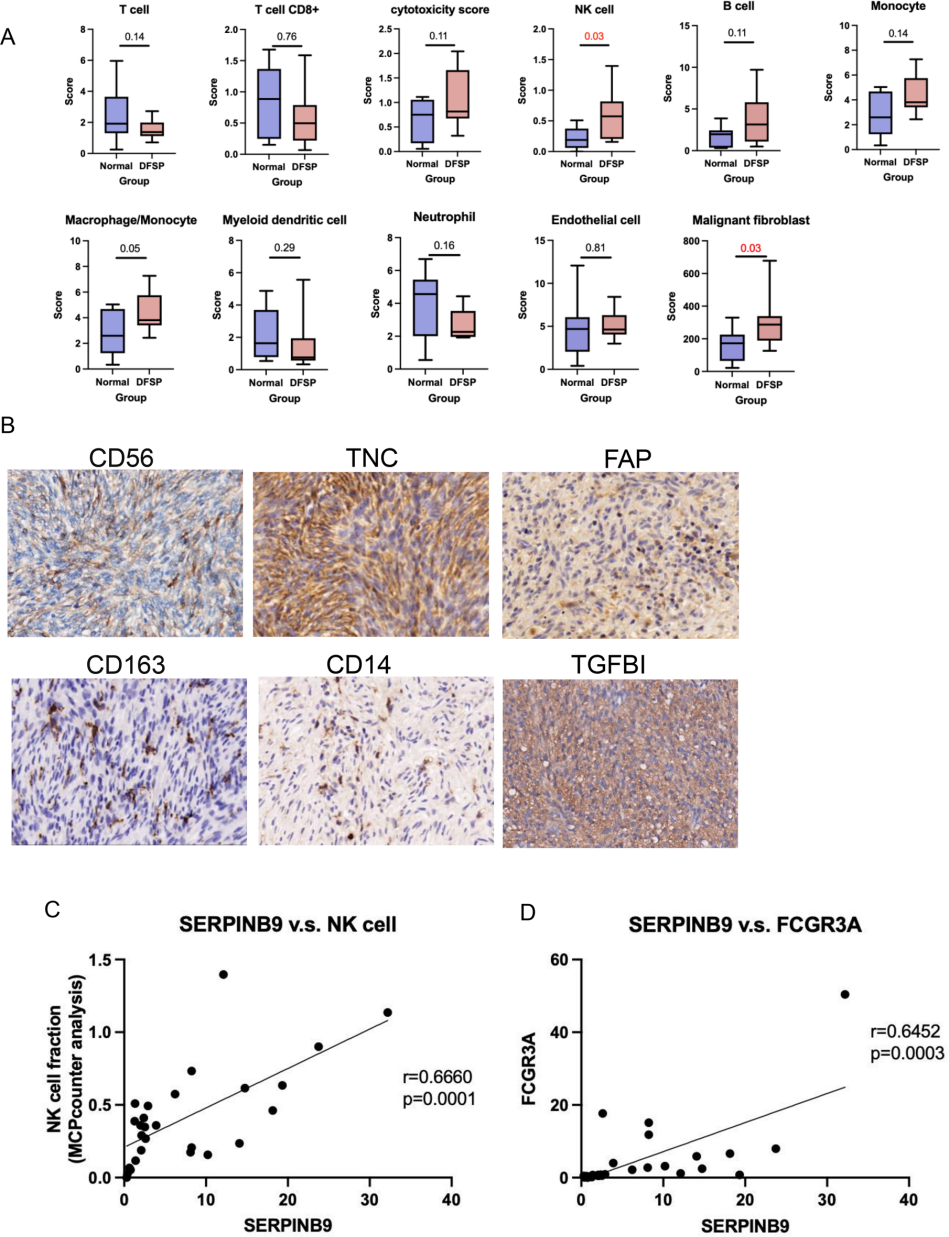
NK cells and tumor-associated macrophage (TAMs) might play a role in DFSP tumor microenvironment. (**A**) Abundance of the tumor microenvironment cell proportions in DFSP and normal adjacent tissues quantified by MCP counter algorithm. Data were obtained from DFSP transcriptomic study by Xiao Zhang et al. [11]. (**B**) CD56 shows weak staining in DFSP tumor tissues. Immunohistochemistry stain of CD14, CD163 and TGFBI in DFSP tumor tissue, which relate to the TAMs. Tenascin C (TNC) and fibroblast activation protein-α (FAP), biomarkers for the malignant fibroblasts, are diffusely positive in DFSP tissues. (400×, patient #1). (**C-D**) Correlations between *SERPINB9* and abundance of NK cell fraction (**C**), *FCGR3A* (**D**) in DFSP tissues. Data were obtained from DFSP transcriptomic study by Xiao Zhang et al. [11]. Abundance of NK cell fraction and *FCGR3A* mRNA level were calculated by MCP-counter algorithm

To delineate the immune landscape of DFSP, we first deployed the Microenvironment Cell Population (MCP)-counter algorithm for a bioinformatic estimation of immune cell infiltration based on public transcriptomic data [11]. This analysis reveals distinct cell abundance profiles in DFSP tumors compared to adjacent normal tissues (Fig. 8A), with significantly elevated predicted fractions of natural killer (NK) cells and malignant fibroblasts in tumors.

To experimentally validate these findings, we performed immunohistochemistry (IHC) on DFSP tissue samples. The results corroborated the bioinformatic predictions: CD56 (an NK cell marker) shows localized weak staining, while markers for monocytes/macrophages (CD14, CD163) and a secretory protein mainly secreted by M2-like tumor-associated macrophage (TAM), TGFBI, are positive in the tumor mesenchyme. Biomarkers for malignant fibroblasts (TNC, FAP) are also diffusely positive (Fig. 8B). Furthermore, to explore potential interactions within the same transcriptomic profile, we analyzed the correlation between SERPINB9 expression and NK cell abundance using the MCP-counter algorithm on the aforementioned dataset [11]. This further analysis demonstrates a strong positive correlation between SERPINB9 mRNA levels and the predicted NK cell fraction (*r* = 0.6660, *p* = 0.0001), as well as with FCGR3A (encoding CD16, another key NK cell marker) (*r* = 0.6452, *p* = 0.0003) (Fig. 8C–8D).

## Discussion

DFSP is generally regarded as a low-grade malignancy. Nevertheless, approximately 10–20% of cases exhibit fibrosarcomatous transformation. This transformation, FS-DFSP, when present in more than 5% of the tumor volume, is associated with an increased risk of local recurrence and distant metastasis [1, 2, 12]. FS-DFSP can develop from classic DFSP or occur *de novo*. For the unresectable, locally advanced or metastatic DFSP, conventional cytotoxic chemotherapy and immunotherapy have generally been ineffective for DFSP [1]. Imatinib is the first-line post-surgery treatment and has produced response rates of 50% to 60%, but there is no established salvage treatment following imatinib failure [13, 14]. While conventional immune checkpoint inhibitors have shown promising results in treating various types of skin cancers, including melanoma, cutaneous squamous cell carcinoma and Merkel cell carcinoma, they show limited efficacy in DFSP. Beyond common reasons for poor response to immune checkpoint inhibitors- such as low tumor mutational burden, insufficient immune cell infiltration, or lack of PD-L1 expression- the heterogeneity in DFSP tumor biology and the *COL1A1-PDGFβ* fusion gene in DFSP may impede the effectiveness of immune checkpoint inhibitors, as the fusion gene can create an immunosuppressive tumor microenvironment (TM) [15]. Therefore, employing high-throughput sequencing to thoroughly investigate the pivotal drivers of DFSP tumorigenesis could uncover potential novel therapeutic targets, especially immune target for the treatment of DFSP.

Notably, in this study more proteins were identified in DFSP tumors than in matched healthy skin. This likely stems from the tumor’s greater biological complexity, such as cellular heterogeneity, an active microenvironment, and dysregulated expression, even with equivalent tissue amounts and standardized protocols. Our key conclusions are based on quantitative differential expression, not total protein counts.

Our proteomic analysis has disclosed an enrichment of the spliceosome pathway in DFSP tumor tissues, with a particular emphasis on the significant overexpression of a key mRNA splicing factor, SF3B. SF3B is an essential component of the U2 snRNP complex of the spliceosome, playing a critical role in the early stages of pre-mRNA splicing. Dysregulation of selective splicing can accelerate cancer progression by influencing various aspects of cancer biology, including cancer cell proliferation, metabolism, angiogenesis, tumor metastasis, and programmed cell death. Mutations in SF3B may be a driving force in tumorigenesis. Moreover, alterations in SF3B expression, not only mutations, may also play a crucial role in the development and progression of cancer [16].

In recent years, some small molecule inhibitors targeting SF3B1 have been identified. Studies have reported that SF3B1 is highly expressed in various cancers, such as breast cancer, liver cancer, prostate cancer and endometrial cancer. Its expression is closely associated with cancer invasiveness and prognosis [17–19]. Overexpression of SF3B1 in prostate cancer is associated with the malignancy of the tumor. Suppressing SF3B1 expression has been shown to decrease the invasiveness of prostate cancer cells, suggesting that SF3B1 may serve as a novel prognostic biomarker and a potential therapeutic target for prostate cancer [20].

Our study found that the SF3B and SerpinB9 were significantly upregulated in DFSP tumors, suggesting that dysregulation of the spliceosome pathway may contribute to the malignant progression of DFSP. To investigate the correlation between SF3B and SerpinB9, we discovered that the splicing of SerpinB9 was affected by SF3B complex in DFSP. Knockdown of *SF3B1* and *SF3B2* markedly suppressed the expression of *SERPINB9* but did not affect the level of pre-*SERPINB9* in DFSP cells. Consistently, treatment with PLB, the specific inhibitor of SF3B1, also significantly downregulated mature *SERPINB9* expression in FS-DFSP cells without altering pre-*SERPINB9* levels. These results indicate that SF3B1/2-mediated splicing is required for the splicing of *SERPINB9* mRNA in DFSP, and that SerpinB9 expression is regulated at the post-transcriptional level through the spliceosome pathway.

It has been reported that *SERPINB9* is commonly amplified and highly expressed in cancer cells, and its overexpression correlates with a poor response to immune checkpoint blockade [21]. SerpinB9 acts as an inhibitor of GrB, a serine protease that plays a critical role in the cytotoxic function of immune cells such as CTLs and NK cells. Under physiological conditions, SerpinB9 primarily functions to protect immune cells from being killed by GrB. However, some cancers exploit its production to evade immune-mediated cell death [22]. Consequently, SF3B may function as an upstream regulator of SerpinB9, and targeting SF3B may represent a potential therapeutic approach to suppress SerpinB9-mediated malignant behaviors in DFSP, especially in the FS-DFSP.

SerpinB9 is also related to the TAMs and the cancer-associated fibroblasts (CAFs). Overexpression of SerpinB9 in cancer cells can facilitate immune evasion by shielding these cells from GrB-mediated apoptosis. Both CAFs and TAMs are integral components of the TM and play pivotal roles in cancer progression and immune evasion. CAFs can promote immune evasion through various mechanisms, including remodeling the extracellular matrix, secreting immunosuppressive factors, and modulating immune cell infiltration and function. TAMs, often exhibiting a pro-tumoral and immunosuppressive phenotype within the TM, can suppress anti-tumor immune response by secreting immunosuppressive cytokines and expressing immune checkpoint molecules. SerpinB9 may interact with CAFs and TAMs to enhance immune evasion [23]. For instance, cancer cells expressing high levels of SerpinB9 might be more resistant to the cytotoxic effects of immune cells, thereby aiding their survival in the immunosuppressive TM orchestrated by CAFs and TAMs. However, the specific interactions between SerpinB9, CAFs, and TAMs, as well as their collective impact on immune evasion, are intricate and highly likely depending on the cancer type and the unique characteristics of the TM. Further research is essential to fully elucidate these interactions and their implications for cancer therapy.

Suppressive immune cells, such as TAM and Treg, have been found to highly express SerpinB9 within the ECM of some cancers [24]. Among the ECM glycoproteins that were upregulated, TGFBI and TNC were shown significant increases in DFSP samples. TGFBI is predominantly secreted by M2-polarized TAMs in the TM. As an ECM-associated secretory protein, TAM-derived TGFBI has been reported to mediate tumor-stromal crosstalk, enhance cell motility and invasiveness, and foster an immunosuppressive microenvironment that facilitates tumor progression [25, 26]. We also identified that markers of TAM including CD163 and CD14, expressed in stromal of DFSP tumors, indicating that TGFBI–TAM axis may contribute to local invasion and disease progression in this malignancy. As a key ECM glycoprotein, TNC remodels the TM and enhances cell motility, supporting its contribution to DFSP progression. Recently, a novel TNC-PDGFD fusion in a case of FS-DFSP was reported [27].

In our data, the SerpinB9 was found to be overexpressed in DFSP tumors and shown a positive correlation with the NK cells, indicating a complex interaction between immune system and tumor cells. One possibility is that the tumor cells may utilize SerpinB9 as a mechanism to modulate the immune response. It might interfere with NK cell function in a way that could be a strategy for the tumor to evade or dampen NK cell-mediated attacks. Another explanation could be the existence of a complex interplay between TM and the immune system. The overexpression of SerpinB9 might be a consequence of certain signaling pathways or factors within the tumor that also influence NK cell behavior, creating an apparent positive correlation. It is also possible that SerpinB9 is involved in feedback loops or regulatory mechanisms that are activated in response to NK cell activity, leading to its upregulation while also influencing NK cell numbers or functionality. Furthermore, our study also showed that in the same DFSP patients, SerpinB9 is more expressed in the fibrosarcomatous parts of the tumors than in the classic parts. These findings suggest that there may be specific cancer subtypes or contexts where unique molecular events drive both the overexpression of SerpinB9 and the modulation of NK cells in a coordinated manner.

SerpinB9 had been found to play a role in tumor drug resistance, as well as sustaining cancer stemness, suggesting that it could be a potential target for cancer therapy [28]. FS-DFSP patients, who are at higher risks of metastasis and death, often require targeted drug therapy. However, imatinib usually shows drug resistance after being used for half a year to one year, leading to significant challenges in subsequent treatments. A novel immune therapy targeting SerpinB9 has been reported to successfully attenuate the malignant melanoma in both cell and mouse models [29]. The small-molecule inhibitor of SerpinB9 could target cancer cells, CAFs and TAMs simultaneously, and the deletion of SerpinB9 in both the tumor and host significantly suppresses tumor growth. The high expression of SerpinB9 in FS-DFSP may be of assistance in exploring potential alternative therapeutic strategies for these patients.

Additionally, the SerpinB9 expression is likely to correlate with the extent of tumor differentiation, staging and the overall survival, serving as an independent predictor of prognosis in patients with hepatocellular carcinoma, as reported in previous studies [30]. The IHC data of our research also showed the higher expression of SerpinB9 in FS-DFSP. The DFSP is a relatively rare sarcoma, given the tumor heterogeneity of DFSP, we assessed SerpinB9 expression in different subtype regions from the same patient to minimize bias. Nevertheless, the relationship between SerpinB9 and disease prognosis requires long-term follow-up in a larger, diverse patient cohort. In FS-DFSP tumor cells, treatment with the SF3B1 inhibitor could downregulate the SerpinB9 gene expression in vitro, consistent with a previous study that the *SERPINB9* mRNA level is affected by the SF3B component (Additional file 3) [31]. However, the pre-*SerpinB9* mRNA, has not been affected by SF3B1 inhibition, suggesting that SF3B1-mediated RNA splicing might be one of the driving factors behind SerpinB9 expression and tumor transformation from classic type to fibrosarcomatous type.

This study still has several limitations. Firstly, due to the rarity of DFSP and its clinical similarity to benign tumors, fresh tumor specimens were extremely difficult to obtain. Therefore, only five cases were included for proteomic analysis. Secondly, we failed to establish a link between SF3B and SerpinB9 in vivo model, due to a lack of well-established animal model for DFSP. Thirdly, the specific role of SerpinB9 has not been fully elucidated, future studies are needed to explore the potential regulatory relationship between SerpinB9 and immune cells, especially NK cells. Fourthly, the therapeutic potential of anti-SerpinB9 still need further investigation both in vitro and in vivo. Finally, the low abundance of NK cells in DFSP primary cells precluded in vitro experiments to validate the causal link between SerpinB9 and NK cells.

In conclusion, comparative proteomics studies are essential for understanding the molecular mechanism of cancer and for biomarker discovery, especially for rare tumors. Our results revealed the involvement of the spliceosomal components SF3B regulating an immune factor *SERPINB9* mRNA splicing in DFSP. The modulation of the NK cells and TAMs in TM may contribute to the development of DFSP. These findings suggest new avenues for therapeutic intervention and aid in the identification of potential tumor markers and understanding of the mechanisms of DFSP.

## Electronic supplementary material

Below is the link to the electronic supplementary material.

Supplementary Material 1

Supplementary Material 2

Supplementary Material 3

Supplementary Material 4

## Data Availability

The mass spectrometry proteomics data have been deposited to the ProteomeXchange Consortium via the PRIDE partner repository with the dataset identifier PXD074336. Other data supporting the findings of this study are available from the corresponding author upon reasonable request.

## Abbreviations

DFSP: Dermatofibrosarcoma protuberans
FS-DFSP: Fibrosarcomatous DFSP
ANT: Adjacent non-tumor
HDF: Human dermal fibroblasts
PLB: Pladienolide B
TNC: Tenascin C
FAP: Fibroblast activation protein-α
TGFBI: Transforming growth factor-β induced
SerpinB9: Serine protease inhibitor B9
DEP: Differentially expressed proteins
IHC: Immunohistochemistry
RT-qPCR: Real-Time Quantitative PCR
PPI: Protein-protein interaction
BPS: Branch point sequence
FFPE: Formalin-fixed paraffin-embedded
CAF: Cancer-associated fibroblasts
TM: Tumor microenvironment
IT: Injection time
