# Supplementary material for: Proteomics-based approach reveals the involvement of spliceosomal components SF3B and SerpinB9 in dermatofibrosarcoma protuberans
Source: Orphanet J Rare Dis. 2026 Jun 26;21:267. doi: 10.1186/s13023-026-04458-4 (PMC13449583; doi:10.1186/s13023-026-04458-4)
Supplement: Supplementary file 1 [file 13023_2026_4458_MOESM1_ESM.docx]

Additional file 1. Detailed clinical and histologic characteristics of patients’ population.

| Characteristics | Gender | Age | Tumor location | Pathological type | Operation | Sample |
| --- | --- | --- | --- | --- | --- | --- |
| Patient #1  Patient #2  Patient #3  Patient #4  Patient #5  Patient #6  Patient #7  Patient #8  Patient #9  Patient #10  Patient #11  Patient #12  Patient #13  Patient #14  Patient #15  Patient #16  Patient #17  Patient #18  Patient #19  Patient #20  Patient #21  Patient #22  Patient #23  Patient #24  Patient #25  Patient #26  Patient #27  Patient #28  Patient #29  Patient #30  Patient #31  Patient #32  Patient #33 | male  male  male  male  male  female  female  male  male  male  male  female  male  female  male  male  male  female  female  male  male  female  female  male  male  female  female  male  male  female  male  male  male | 43  33  64  48  25  26  28  34  34  31  27  21  44  33  52  32  55  56  27  25  24  34  43  30  31  28  37  33  48  22  60  33  41 | back  back  back  back  back  abdomen  back  back  left clavicular region  abdomen  abdomen  back  left upper arm  left shoulder  abdomen  right shoulder  left thigh  right foot  chest  scalp  back  abdomen  right groin  abdomen  left clavicular region  chest  forehead  abdomen  left forearm  forehead  right neck  abdomen  right shoulder | classic  classic  classic  classic  classic  classic  classic  classic  classic  classic  classic  classic  classic/fibrosarcomatous  classic/fibrosarcomatous  classic/fibrosarcomatous  classic/fibrosarcomatous  classic/fibrosarcomatous  classic  classic  classic  fibrosarcomatous  fibrosarcomatous  fibrosarcomatous  classic  classic  classic  classic  classic  fibrosarcomatous  fibrosarcomatous  fibrosarcomatous  fibrosarcomatous  fibrosarcomatous | Proteomic analysis/IHC/qPCR  Proteomic analysis/IHC/qPCR  Proteomic analysis/IHC/qPCR  Proteomic analysis/IHC/qPCR  Proteomic analysis/IHC/qPCR  WB/qPCR  WB/qPCR  WB/qPCR  qPCR  qPCR  qPCR  qPCR  IHC/qPCR  IHC/qPCR  IHC/qPCR  IHC/qPCR  IHC/qPCR  Cell viability/qPCR/siRNA  Cell viability/qPCR/siRNA  Cell viability/qPCR/siRNA  qPCR/siRNA  qPCR/siRNA  qPCR/siRNA  qPCR  qPCR  qPCR  qPCR  qPCR  qPCR  qPCR  qPCR  qPCR  qPCR | tumor and ANT tissues  tumor and ANT tissues  tumor and ANT tissues  tumor and ANT tissues  tumor and ANT tssues  tumor and ANT tissues  tumor and ANT tissues  tumor and ANT tissues  tumor and ANT tissues  tumor and ANT tissues  tumor and ANT tissues  tumor and ANT tissues  tumor and ANT tissues  tumor and ANT tissues  tumor and ANT tissues  tumor and ANT tissues  tumor and ANT tissues  DFSP primary cells  DFSP primary cells  DFSP primary cells  DFSP primary cells  DFSP Primary cells  DFSP primary cells  DFSP primary cells  DFSP primary cells  DFSP primary cells  DFSP primary cells  DFSP primary cells  DFSP primary cells  DFSP primary cells  DFSP primary cells  DFSP primary cells  DFSP primary cells |
