# Supplementary material for: Proteomics-based approach reveals the involvement of spliceosomal components SF3B and SerpinB9 in dermatofibrosarcoma protuberans
Source: Orphanet J Rare Dis. 2026 Jun 26;21:267. doi: 10.1186/s13023-026-04458-4 (PMC13449583; doi:10.1186/s13023-026-04458-4)
Supplement: Supplementary file 2 [file 13023_2026_4458_MOESM4_ESM.docx]

**Additonal file 1.** Detailed clinical and histologic characteristics of patients’ population.

**Additonal file 2.** Differentially expressed genes (|Fold Change| > 1.5, FDR < 0.05) detected from quantitative proteomics data.

**Additional file 3.** *SERPINB9* mRNA level was visualized using NCBI GEO2R webtool. Expression data of each sample was obtained from GSE157725 datasets. (A) *SERPINB9* mRNA level of PC3 cells treated with control siRNA (siCTR) or SF3B2 siRNA(siSF3B2). (B) *SERPINB9* mRNA level of PC3 cells treated with DMSO (CTR) or PLB.
